# Supplementary material for: Bayesian refinement of protein structures and ensembles against SAXS data using molecular dynamics
Source: PLoS Comput Biol. 2017 Oct 18;13(10):e1005800. doi: 10.1371/journal.pcbi.1005800 (PMC5662244; doi:10.1371/journal.pcbi.1005800)
Supplement: S1 Archive — (BZ2) [file pcbi.1005800.s011.tar.bz2 › gromacs_4.62_bayesian_saxs_refi/share/html/online/ene.html]

ene file format


## ene file format

|  |  |
| --- | --- |
| Main Table of Contents | **VERSION 4.6** |
| GROMACS homepage | **Sat 19 Jan 2013** |

---

### Description

The ene file extension stands for binary energy file. It holds the
energies as generated during your mdrun.
  
The file can be transformed to a portable energy file (portable
accross hardware platforms), the
.edr file using the program 
eneconv.

See also g\_energy.

---

http://www.gromacs.org
